# Multifaceted polarization and information reliability in climate change discussions on social media platforms

**DOI:** 10.1098/rsos.241974

**Published:** 2025-11-26

**Authors:** Aleix Bassolas, Joan Massachs, Emanuele Cozzo, Julian Vicens

**Affiliations:** ^1^ Eurecat, Centre Tecnològic de Catalunya, Barcelona, Spain; ^2^ Departament de Física de la Matèria Condensada and Institute of Complex Systems (UBICS), Universitat de Barcelona Facultat de Fisica, Barcelona, Spain; ^3^ CNSC-IN3, Universitat Oberta de Catalunya, Barcelona, Spain

**Keywords:** climate change, social networks, polarization, information dynamics

## Abstract

Social media platforms like YouTube and Twitter play a key role in disseminating both reliable and unreliable information about climate change. This study analyses the topology of interactions in Twitter and their relation to cross-platform sharing, content discussions and emotional responses. We examined climate change discussions across four topics: the 27th United Nations Climate Change Conference, the Sixth Assessment Report of the United Nations Intergovernmental Panel on Climate Change, Climate Refugees and Doñana Natural Park. While retweets reinforce in-group cohesion in the form of echo chambers, inter-group exposure is significant through mentions, suggesting that exposure to opposing views intensifies polarization, rather than mitigates it. Ideological divides feature content differences accompanied by steeper negative sentiments, especially from right-leaning communities prone to share low-reliability information. We identified a topological and thematic alignment between platforms, indicating that ideological communities are interconnected across them. Our findings show that climate change polarization is multifaceted, involving ideological divides, structural isolation and emotional engagement. These results suggest that effective climate policy discussions must address the emotional and identity-driven nature of public discourse and seek strategies to bridge ideological divides.

## 1. Introduction

Polarization has become a defining feature of contemporary political and social life. While a certain level of conflict and disagreement is essential to the functioning of democracies, an excessively polarized social environment can detrimentally affect their self-governing capacity [1], particularly when it pertains to issues like climate change that require broad consensus and coordinated action. In this paper, we examine polarization in climate change discourse on Twitter (currently X) and YouTube, using a multidimensional approach. To capture different social and geographical contexts, we analyse four datasets related to milestone events: the 2022 UN Climate Change Conference (COP27), the release of the Sixth Assessment Report by the Intergovernmental Panel on Climate Change (IPCC), the emergent issue of Climate Refugees and a politically charged local debate in Spain over environmental regulation in Doñana.

The term polarization is used across disciplines to describe the division of a population into two well-separated and opposing groups. It can be understood both as a state—the extent to which opinions or characteristics are opposed at a given time—and as a process—the movement towards greater extremes over time [2]. Furthermore, polarization has a specific meaning related to the distribution of opinions, beliefs, attitudes or other measurable attributes in a population (such as wealth or income) [3]. Operationalizations of this concept emphasize the formation of distinct groups in the distribution, with high homogeneity within the group and heterogeneity between groups. The core notion is that polarization is high when a distribution is multi-modal and maximal when it is bimodal (i.e. when there are multiple distinct ‘peaks’ corresponding to social groups) rather than a continuum [4].

Esteban & Ray [3] frame polarization in terms of ‘identification’ and ‘alienation’. Polarization is conceptualized as the result of people strongly identifying with others in their own group while feeling alienation (distance or hostility) towards those in other groups. A formal model is proposed, where society is more polarized when it contains relatively cohesive subgroups that are far apart from each other. High identification within groups means that each of the groups of individuals has internal similarity or solidarity, and high alienation means there is strong effective distance or antagonism between those groups. This idea leads to well-defined mathematical measures in which polarization is essentially the weighted sum of pairwise ‘antagonisms’ between individuals, increasing when people cluster into separated groups.

The shift of political views towards extreme positions and the formation of separate cohesive groups often is related to the presence of echo chambers, where interactions predominantly occur among individuals who share similar beliefs and ideas [5–7]. As interest in measuring [6,8–11] and modelling [12] the dynamics of polarization and echo chambers grows, efforts to identify the factors influencing the interaction patterns face significant challenges. The recommendation algorithms employed by these platforms influence these dynamics [13–15] by limiting exposure to opposing viewpoints in users’ feeds [16]. Yet, the rise of polarization is not only an effect of homophily, as it has been shown that individuals are often exposed to opposed opinions [17].

While polarization is a natural aspect in pluralistic societies, its intensification over the last decades is a matter of debate both among academics and in public discourse [2,18–20]. Multiple studies point to the fact that the widespread adoption of social media has driven an increase in polarization as observed across experimental settings [21] and various platforms [8,16,22]. However, its presence and magnitude vary across subjects, can be triggered by external events [23–26] and is steeper around political discussions than in more general subjects [27]. Platforms also play a role; while polarization has been observed on Twitter, YouTube or Instagram [28–31], in others, such as WhatsApp, there is a depolarization effect [32]. Even though the interest in measuring social and political polarization dates from before the digital era [33], the widespread use of online social media and data accessibility sparked the development of frameworks to quantify it. The techniques developed vary depending on the perspective; while the topology of interactions provides insights into the preferences to form social bonds based on political views [34], textual analysis can capture the divergence in emotions and policies [35].

The combination of a polarized environment [36] and behavioural factors, such as confirmation bias [37,38], has been closely associated with the dissemination of low-reliability information [39], influencing the outcome of presidential elections [40] and increasing vaccine hesitancy [41]. Despite these challenges, there have been concerted efforts to combat the spread of fake news [42]. Nonetheless, debates persist regarding the distinction between reliable and unreliable news cascades. Although initial studies highlighted structural differences [43], subsequent analyses have suggested that disparities may be more attributable to differences in cascade size [44].

Polarization manifests across political issues, but also other kinds of events and subjects as we have witnessed an increase in climate change discussions and related events [20,26,45,46]. Previous research has focused on the origins of this polarization [47], its relation to political affiliations [48] and the influential roles played by media outlets [49] and corporations [50]. Recently, an increasing polarization has been reported in locations affected by extreme weather events or environmental disputes [46]. The sentiment towards climate change mitigation measures varies significantly across regions, shaped by local political and cultural contexts [51]. While the spread of low-reliability information in these contexts has been noted, its connection with polarized environments remains unclear [52–54]. Our study addresses this gap by studying a highly localized and politically charged debate over environmental protection in Spain, Doñana, where the interplay between misinformation, partisanship and institutional trust is especially salient.

The broad concept of polarization can then be further specified, with particular relevance to our work, by focusing on the notions of ideological and affective polarization. Ideological polarization usually refers to the distance between political preferences, identities or parties within a society [2]. In relation to this, the analysis of a retweet network relies on the empirical and theoretically supported assumption that the way users share information is related to and a source of their collective political identity [55,56]. In the same line, information sharing is related to the practice of networked gatekeeping that puts in relation elite and mass polarization [57] and leads to the formation of affective publics [58]. Affective polarization is also emphasized in political science and is distinct from policy disagreement. It describes the phenomenon of partisans feeling not just different, but actively antagonistic towards each other [19,59]. In other words, it is the polarization of feelings or attitudes towards the social groups representing the other, rather than towards policy issues themselves. This is well captured through expressions of antagonism between groups, such as emotional tone in communication. In relation to the structural polarization metrics we apply in this work, it is central to the notion of how users use retweet and mention as different ways of signalling in- and out-group relations and identities [60].

The initial metrics to quantify the macroscopic level of structural polarization primarily concentrate on modularity and the analysis of community boundaries [34,61,62]. However, those only provide a partial perspective on the organization of social connections, as they largely disregard the multi-scalar organization of networks and the role of individual nodes. Metrics designed to capture long-range structural properties include label propagation [63], centrality measures and random walk diffusion [64]. Even though most assume a dipole organization, recent developments can capture multipole polarization [65]. Structural measures in dealing with the networked organization of social relations [66] provide a measure of polarization being content-agnostic. Natural language processing techniques are essential for assessing the content of the divergence on policy-related issues [32,45] and evaluating the emotions of individuals and their interactions [60]. While our approach focuses on structural and affective dynamics at a group level, recent studies have aimed to integrate these with individual-level opinions [31], providing further context into the structural organization of different ideological perspectives.

Building on previous literature that tends to isolate structural, ideological or affective polarization, this study offers an integrated approach to capture their intersection in climate change discourse. Drawing on Twitter and YouTube data, we examine how community structure, emotional tone and media reliability interact across platforms and contexts. To do so, we combine network analysis (to assess structural patterns), ideological measures (using ideological bias and source reliability) and sentiment metrics (focused on toxicity in quote tweets and cross-group mentions). This multidimensional approach allows us to observe how different dimensions of polarization reinforce one another. Users with similar ideological leanings tend to cluster in retweet networks and express aligned affective reactions, particularly in politically salient debates. Interactions across communities, while rare in the form of retweets, are more frequent through mentions and often carry antagonistic sentiment—suggesting emotional confrontation despite low structural connectivity.

Our main contribution is the development of an integrative framework that captures structural, ideological and affective polarization across digital platforms. Applying this framework to four climate-related events, we examine how polarization manifests differently depending on the topic and geographical context, ranging from global summits to locally contentious environmental debates. This multidimensional analysis reveals consistent links between the structure of online communities, the emotional tone of user interactions, and the reliability of the content being shared. Notably, we observe that polarization is not confined to a single platform: ideologically aligned groups on Twitter also engage with similar content on YouTube, indicating a broader cross-platform coherence. These findings underscore the importance of moving beyond single-platform or single-dimension studies in order to understand the full complexity of polarization in digital climate discourse.

## 2. Data description

We extracted four datasets related to climate change topics from Twitter through the Twitter Academic API. These datasets correspond to the 2022 United Nations Climate Change Conference (COP27), to the release of the Sixth Assessment Report by the Intergovernmental Panel on Climate Change (IPCC), to the climate refugee crisis resulting from extreme weather conditions (Climate Refugees) and to legislation affecting the natural reserve of Doñana in the south of Spain (Doñana). Tweets were collected using Twarc [67] by searching for keywords related to each discussion. The keywords selected were closely related to each event. For COP27, the keywords included COP27 and related concepts such as *LossAndDamage*. For the IPCC, the keywords were IPCC and its official account (@IPCC_CH). For Climate Refugees, the keywords were related combinations that included *climate refugee(s)* and *climate migration*. For Doñana only the keyword Doñana was used. The datasets for specific events (COP27 and IPCC) were filtered within the span of the pre- and post-event dates. The Climate Refugees and Doñana datasets span a longer period compared to the IPCC and COP27 datasets. The IPCC dataset, in particular, covers only nine days, coinciding with the peak discussion surrounding the release of the Sixth Assessment Report (see table 1). We extracted the corresponding YouTube dataset by analysing the URLs referenced in each Twitter dataset and downloading the posts and comments using the YouTube Data API. To ensure relevance, we filtered the posts by searching for occurrences of specific keywords in each video title or description (electronic supplementary material, §S1).

**Table 1.** Summary table of the Twitter datasets analysed. Minimum and maximum dates, observed number of users and number of tweets by typology.

	min. date	max. date	users	tweets	retweets	replies	quotes
COP27	01-09-2022	27-11-2022	1 351 903	866 753	4 977 874	205 973	175 078
IPCC	18-03-2023	26-03-2023	157 056	31 138	267 971	45 863	7 751
C. Refugees	10-03-2008	31-12-2022	841 454	384 267	1 376 057	139 699	38 909
Doñana	01-01-2019	30-04-2023	290 782	139 478	1 187 646	135 245	25 056

Unlike the Twitter dataset, the period of the comments is longer due to users frequently referencing older videos. Each dataset exhibits peaks at different times based on its unique characteristics. In particular, we observe a strong alignment in the IPCC and COP27 datasets with the event dates (table 2). Additional insights, including the data collection procedure, can be found in electronic supplementary material, §S1.

**Table 2.** Summary table of the YouTube datasets analysed. Number of videos in each community, minimum and maximum dates observed, and average number of views, likes and comments.

	videos	min. date	max. date	avg. views	avg. likes	avg. comments
COP27	624	11-02-2008	12-05-2023	571 964.35	5 860.32	306.99
IPCC	145	03-11-2007	09-06-2023	350 178.66	8 991.67	1 455.16
C. Refugees	215	22-11-2009	06-11-2023	243 720.27	3 604.81	527.40
Doñana	191	11-01-2009	01-06-2023	101 098.32	3 622.68	246.25

## 3. Results

### 3.1. Network analysis and structural polarization

**Figure 1.**
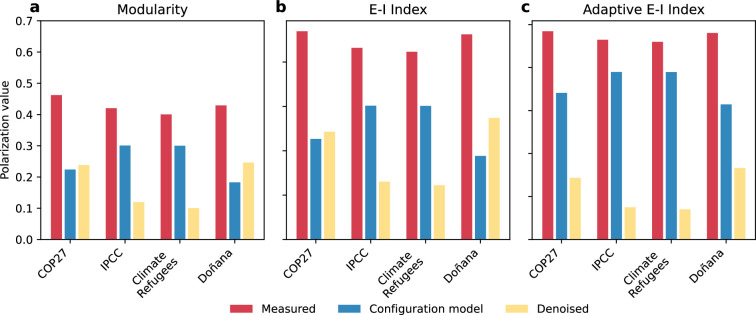
Polarization in the Twitter dataset. Quantification of the polarization in Twitter: (a) modularity, (b) reversed *E*–*I* index, and (c) reversed adaptive *E*–*I* index. We provide the measures for the observed networks in red, across 100 realizations of the configuration model with preserved degree sequences in blue, and the difference between them (denoised value) in yellow.

We evaluate the structural polarization on Twitter by considering the unweighted network of retweets with undirected links indicating a retweet interaction between two users. Previous studies have used retweets as a proxy for the influence between individuals, allowing for the computation of their latent ideology [45,63]. Similarly, we aim to measure how the influence between individuals shapes network organization. We applied the Metis partition algorithm [68] to split the graph into two clusters, and we calculated the modularity [69], the reversed *E*–*I* index [70] and the reversed adaptive *E*–*I* index [48] that adjusts for the cluster sizes. These metrics evaluate the topological interactions between users, the mesoscale structure and the extent to which users in different groups interact with one another without considering the content. The results for the four datasets analysed are presented in figure 1. Following [34], we computed polarization metrics in the observed network Φ(G), as well as in the ensemble of configuration models with equivalent degree distribution $\Phi (G_{CM})$ [71] and calculated the denoised value $\hat{\Phi }(G)=\Phi (G)-\Phi (G_{CM})$. For completeness, we report the standardized values [34] in electronic supplementary material, figure S3.

All networks exhibit structural polarization according to the denoised value, suggesting the presence of two groups of strongly connected individuals. In particular, the COP27 and Doñana datasets displayed larger denoised values, suggesting a stronger polarization around the topics. The higher structural polarization observed in the COP27 dataset may be attributed to transnational spheres using different languages that do not interact with each other [72]. The first and second largest communities, in terms of the number of messages, are dominated by English. The third and fourth communities primarily consist of Portuguese messages, followed by others where French and Spanish are more prevalent. Further details on this distribution per dataset and community can be found in electronic supplementary material, §S2.2. The Doñana dataset captures the discussion around a new law related to natural park preservation, which involves political parties and users with strong political affiliations like politicians, journalists or activists. The lower polarization observed in the IPCC dataset may be due to its nature as a scientific report release rather than a mainstream international conference like COP, potentially attracting a more scientifically educated audience and inducing a lower level of polarization. Although the largest community (labelled as 0) is composed mainly of scientists, primarily from the UK, other communities consist of activists (community 3) or politicians (community 6), such as @GretaThunberg and @BarackObama, respectively.

**Figure 2.**
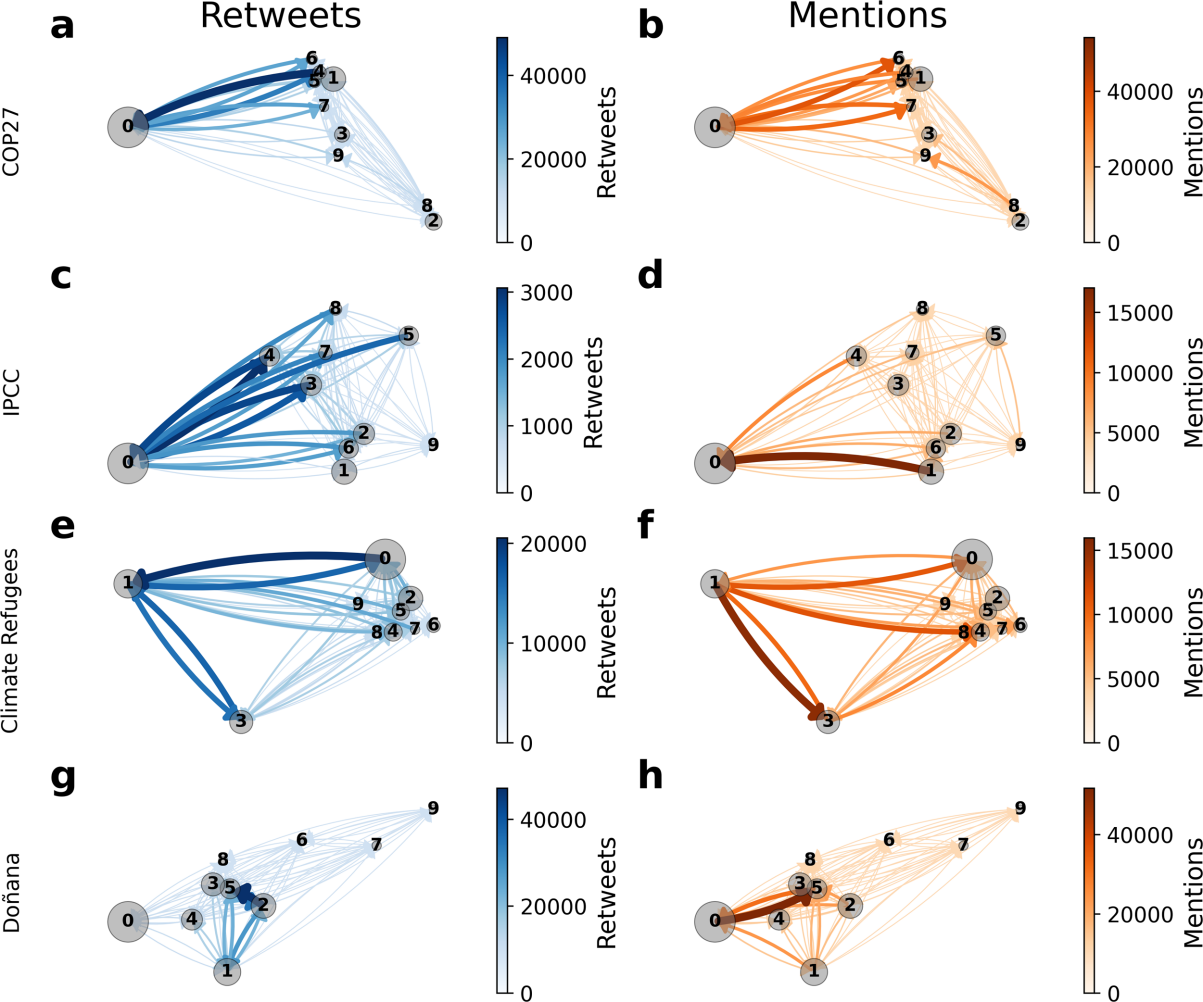
Community interaction network. Interaction between the ten largest communities in (a,b) COP27, (c,d) IPCC, (e,f) Climate Refugees and (g,h) Doñana. We report the interactions via (a,c,e,g) retweets and (b,d,f,h) mentions. Arrows indicate the direction of the interaction, the colour intensity and width, the volume. The size of the dots represents community size. In blue, we show the interaction through retweets and, in orange, through mentions.

While the retweet network has been previously interpreted as the influence between social media users, other types of interactions, such as mentions, often feature different structural properties [61]. We examine the network of retweets and mentions among the 10 largest communities of the networks in figure 2. We identified these communities using modularity optimization on the directed weighted graph of retweets only [69], and then we incorporated mentions. This approach allows us to identify clusters based on influence and later analyse how they interact through mentions and retweets. The interaction patterns among the largest community (labelled as 0) and the remaining communities vary across datasets and types of interactions. In the COP27 and IPCC networks, the smaller communities mostly retweet content from the largest community, which is more isolated and engages in fewer interactions with the rest of the network. The largest communities include official accounts and major figures, such as the Secretary-General of the United Nations (@antonioguterres) or the Intergovernmental Panel on Climate Change (@IPCC_CH), representing COP27 and the IPCC, respectively. Conversely, the Doñana network exhibits higher flow heterogeneity between communities, with community 5 playing a central role in attracting most of the flow. The detailed overview of the users in the community reveals the presence of the president of Spain (@sanchezcastejon), the ruling political party (@PSOE) and sympathizer journalists (@eldiarioes, @iescolar). There are significant structural differences depending on the type of interaction. For example, in the IPCC network, there are relatively few retweets from community 1, with accounts concentrated on flow such as @uksciencechiefUK (Government Office for Science) and @BernieSpofforth (the anti-climate movement figure) to community 0, formed by well-known climate science accounts such as @ed_hawkins or @MrMatthewTodd. However, there is a high volume of mentions between these communities (see details in electronic supplementary material, §S2.1).

#### 3.1.1. Isolation and entropy

We further evaluate the connectivity patterns between communities by quantifying the structural properties of their inter- and intra-group interactions. To quantify the isolation of communities, we calculated the directed interactions between them, and we computed the normalized outflow difference given by


(3.1) $$\Delta F_{i}^{\text{ out}}=\frac{\sum_{j,j\neq i}T_{ij}-T_{ii}}{\sum_{\forall j}T_{ij}},$$

where $T_{ij}$ is the number of interactions from community *i* to community *j*. $\Delta F_{i}^{\text{out}}$ provides a measure of a community’s isolation. A value of −1 indicates that a community has only internal flow, whereas a value close to 1 suggests that most of the flow is directed towards other communities. A value of 0 signifies that half of the flow is external to the community, while the other half remains internal.

To understand the diversity of interactions among communities, we compute the entropy of flows as


(3.2) $$H_{i}^{\text{out}}=-\sum_{\forall j}p_{ij}\log p_{ij},$$

where $p_{ij}$ is the probability of having an interaction from *i* to *j* given by $T_{ij}/\sum_{\forall j}T_{ij}$. While $\Delta F_{i}^{\text{out}}$ quantifies the ratio between external and internal links and goes from −1 (when all flows are internal) to 1 (when all flows are external), the entropy ($H_{i}^{\text{out}}$) provides information on the variety of those flows. The entropy is 0 when there is no variety of flows and 1 when it is maximum. Most of the largest communities exhibit a certain degree of isolation, indicated by negative values. In the COP27 network, the most isolated communities are 2 and 3, both Brazilian ($\Delta F_{2}^{\text{out}}=-0.97$ and $\Delta F_{3}^{\text{out}}=-0.94$), followed by community 1 ($\Delta F_{1}^{\text{out}}=-0.88$), which is the most isolated and has the lowest entropy ($H_{1}^{\text{out}}=0.05$) among English-speaking groups. This community consists of sceptics with influential accounts such as @JamesMelville and @BernieSpofforth. Following the same pattern, in the IPCC network, community 1 has the highest isolation value ($\Delta F_{1}^{\text{out}}=-0.98$) and the lowest entropy ($H_{1}^{\text{out}}=0.014$). This community also includes, among other accounts, @BernieSpofforth. Similarly, in the Climate Refugees network, community 6 is the most isolated ($\Delta F_{6}^{\text{out}}=-0.93$) and has the lowest entropy ($H_{6}^{\text{out}}=0.03$), featuring accounts such as @MaximeBernier and @PrisonPlanet. In the Spanish Doñana network, community 0 is the most isolated ($\Delta F_{0}^{\text{out}}=-0.95$) and has the lowest entropy ($H_{0}^{\text{out}}=0.02$). Across all datasets, communities with right-wing alignment demonstrate strong isolation and low entropy, implying that their messages have limited spread across the network.

However, the pattern changes when considering the mention graph. The largest mainstream communities show far less mentions of other communities; for instance, community 0 on IPCC and COP27 networks has high isolation ($\Delta F_{0}^{\text{out}}=-0.5$ and $\Delta F_{0}^{\text{out}}=-0.57$, respectively). Whereas the low-reliability communities display lower isolation and more external mentions, as is the case of community 1 in COP27 ($\Delta F_{1}^{\text{out}}=0.37$ and $H_{1}^{\text{out}}=0.25$). These results reinforce, first, the heterogeneity in group connectivity depending on the interaction types and, second, different levels of isolation by communities.

### 3.2. Ideological polarization

#### 3.2.1. Bias and reliability

**Figure 3.**
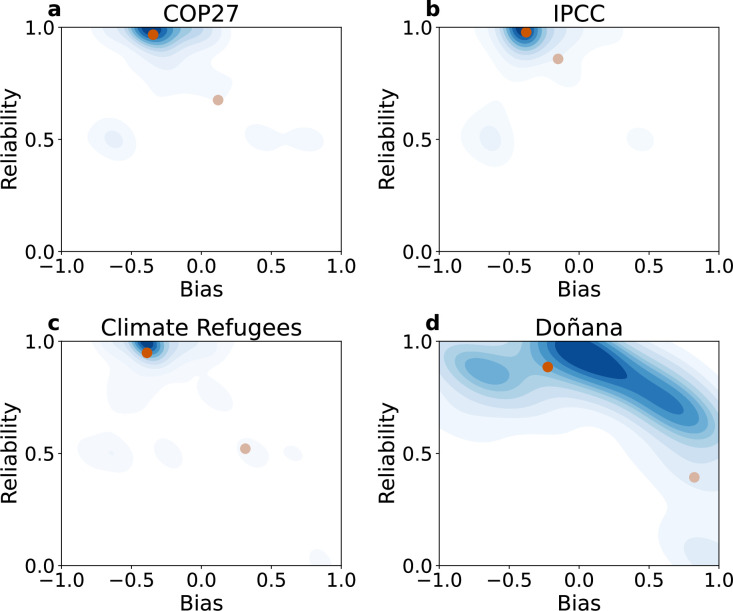
Reliability as a function of ideological bias in the studied networks. Density plot showing the distribution of reliability as a function of bias for the communities detected in (a) COP27, (b) IPCC, (c) Climate Refugees and (d) Doñana. For each dataset, the two communities with the largest and most divergent ideological alignment are identified. In dark brown, the left-biased community; and in light brown, the right-biased community

We assess if the structural polarization is related to the divergence in political views and information diet by calculating the bias and reliability of the URLs shared by the users. Denser connections between users with similar ideological biases could indicate a closer influence between them and a greater disagreement on climate change discussions. Information on the ideological bias and reliability of news outlets was obtained from MediaBias fact-check [73], where positive and negative ideological biases correspond to right-wing and left-wing media, respectively. Outlets with higher reliability are considered more trustworthy and tend to share more verified news. For the Doñana dataset, the reliability and bias were based on a separate dataset [74], given that most media sources are Spanish. To facilitate the analysis, we have normalized the bias within the range [−1, 1] and the reliability within the range [0, 1]. The minimum and maximum biases are set according to the higher absolute value observed. In figure 3a–d, we show the density plot of community reliability as a function of their ideological bias according to the shared URLs in the IPCC, COP27, Climate Refugees and Doñana networks. We computed user-level values by averaging over the sources they shared and then calculated community-level values by averaging over the users. All datasets exhibit a left-wing bias, as most communities show negative values, especially in the IPCC and Climate Refugee networks. Possible explanations include a systemic bias in media coverage of these topics, the tendency of climate change sceptics to share information from unconventional sources that are not explicitly flagged by media bias indicators, or an over-representation of left-wing users in climate change discussions, as they may be more likely to engage due to greater awareness of the issue. The left-wing bias is particularly pronounced in the IPCC and Climate Refugee networks, where the cluster of large communities is centred around negative values. However, the bias is more dispersed in the Doñana dataset, possibly due to differences in the shared media sources or a wider ideological variety.

The communities with right-wing bias we have identified are: community 1 in COP27 (with key actors such as @JamesMelville) and IPCC (with the presence of @BernieSpofforth), community 6 in Climate Refugees (with @MaximeBernier or @PrisonPlanet), and community 0 in Doñana (with @JuanMa_Moreno or @alfonso_ussia). We performed significance tests to compare the bias and reliability of communities, revealing several significant differences. In terms of bias, the most notable differences (*p* < 0.001) are observed in the previously mentioned right-wing biased communities compared to the other communities. Regarding reliability, community 4 in COP27 (with main actors such as @CarolineLucas), community 3 in IPCC (with @GretaThunberg or @CarolineLucas), community 5 in Climate Refugees (@jeremycorbyn) and community 1 in Doñana (@WWFespana and other conservation organizations) show the most significant higher reliability relative to the other communities (see electronic supplementary material, figures S6 and S7).

Based on the previous findings and manual inspection of the communities, we identified the two largest communities with opposing political leanings. In the IPCC and COP27 datasets, community 0 represents the largest community aligned with the mainstream climate change narrative, while community 1 is the primary advocate of a counter-narrative message. However, in the Doñana network, the labels are swapped, as the right-wing biased community has a larger user base. For the Climate Refugees network, most of the top 10 communities exhibit similar bias–reliability values, except for community 6, which stands out with low reliability and a right-wing bias. In this dataset, we designate community 1 as the primary left-wing biased community, as it includes well-known climate change activists such as @MikeHudema or @GretaThunberg and engages in more interactions with the low-reliability community. Our results point out that most communities in climate change discussions share reliable information and have a left-biased alignment. However, there is at least one large community in each dataset on the right-side alignment that shares less reliable information.

These results indicate differences in the network topology depending on the type of interactions. Retweets, which are linked to influence, connect individuals in communities with similar biases. Instead, a higher number of mentions across communities suggests interactions between individuals from opposing sides of the ideological spectrum.

Given that many networks include users from different countries speaking various languages, we also measured polarization by focusing on the two largest communities that speak the same language but have opposing biases, comparing the retweet and mention networks. The results still indicate polarization in the retweet network, with modularity values ranging from 0.07 in the IPCC network to 0.21 in the Doñana network. Even in the mention networks—where structural scores are lower (modularity is 0.05 in COP27, 0.16 in Climate Refugees, and 0.15 in Doñana)—only the IPCC network shows no polarization (modularity = −0.23). These findings further support the observed disparity in network organization based on interaction typology (see electronic supplementary material, §S2.3, for more details).

#### 3.2.2. Echo chambers

Throughout this section, we assess the presence of echo chambers with a focus on the two largest communities exhibiting opposing ideological biases. Our objective is to assess the interaction patterns between users as a function of their bias. We employed an approach developed in [9] to evaluate the existence of echo chambers. For a given user *i* with degree $k_{i}$, we computed its average bias $x_{i}$ and reliability $y_{i}$, along with the average bias and reliability of its neighbourhood, denoted as $\frac{1}{k_{i}}\sum_{\forall j\neq i}A_{ij}x_{j}$ and $\frac{1}{k_{i}}\sum_{\forall j\neq i}A_{ij}y_{j}$, respectively.

**Figure 4.**
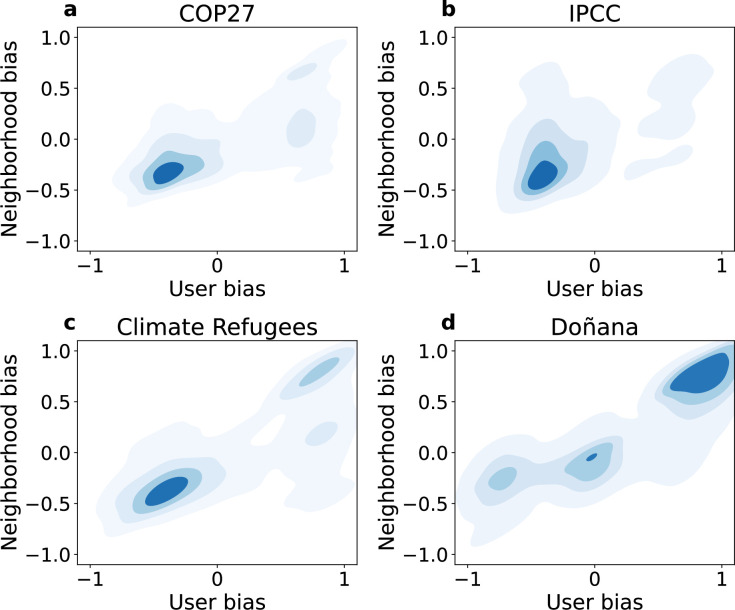
Detection of echo chambers in ideological biases for the retweet networks. Neighbourhood ideological bias as a function of the user bias in the retweet network for (a) COP27, (b) IPCC, (c) Climate Refugees and (d) Doñana.

We show the density plot for the bias of the neighbourhood of users as a function of their values when considering the interactions through retweets in figure 4. As expected from the overall ideological positioning of the users, there is a hotspot on the left of the ideological spectrum and a weaker one on the right side. We have identified a diagonal trend that could be related to the presence of echo chambers. Right-wing biased users interact more with left-wing biased users, as there are two hotspots for positive values in some cases. The presence of echo chambers is more evident in the Doñana dataset than in the rest of the networks, mainly due to the presence of more users with right-leaning discourse. There are three hotspots of comparable magnitude across the diagonal with right-, centre- and left-leaning discourses. Still, the hotspot on the left side of the spectrum displays a lower ideological isolation, since it interacts with users featuring a bias close to 0. The right-wing biased users interact mainly with each other and not with users in the centre. When analysing other types of interactions, such as the mentions, the presence of echo chambers is weaker with nearly horizontal hotspots in the COP27 and IPCC datasets (see details in electronic supplementary material, figure S11). This observation is consistent with the results on polarization from the previous section. The existence of echo chambers is less pronounced when considering different types of interactions, and mentions could serve as a bridge between users with different ideological biases.

We also examine the echo chambers based on the reliability of the content shared by users and their neighbourhoods, where we find three hotspots for high, medium and small values. The hotspots in high reliability suggest that users who share highly reliable sources interact with each other, while low-reliability users interact with users who spread highly reliable sources (see electronic supplementary material, figure S12).

We inspect in detail the user interaction similarity by quantifying their echo chamber [75], without considering the ideology. Given a set of leading users *i*, which have an audience $A_{i}$ composed of the set of users that retweeted it, their chamber $C_{i}$ is the set of users retweeted by audience $A_{i}$. We focus on the two communities with opposing ideological biases, specifically the top 20 users with the most retweets in each of those communities. The first quantity we will analyse is the chamber overlap between a user *i* and a user *j* given by


(3.3) $$q_{ij}=\frac{C_{i}\cap C_{j}}{C_{i}\cup C_{j}},$$

where $C_{i}$ and $C_{j}$ are the chambers of users *i* and *j*. It is worth noting that the quantity $q_{ij}$ is symmetric by definition and is equal to 1 when chambers are identical and 0 when they are completely different.

We compute the chamber overlap distribution $P(q_{ij})$, where a notable disparity between networks is observed (see electronic supplementary material, figure S13). A strong bimodal distribution suggests the presence of echo chambers, as each user has low values of $q_{ij}$ with users outside its echo chamber and high values with those inside it. The results from the COP27 and Climate Refugees datasets suggest that leaders have more heterogeneous chambers compared to the IPCC and Doñana datasets, where we observe a peak for high chamber overlap. The Doñana dataset features a strong echo chamber effect with two separated peaks at low and high values of $q_{ij}$. We have performed hierarchical clustering of the top 20 users per community based on the similarity of their chambers, showing a strong alignment between the communities detected (for more details, see electronic supplementary material, §S2.6).

### 3.3. Policy and affective polarization

On account of the structural polarization observed, with groups of users sharing ideological bias, we further investigate if there are perceptible differences in their climate change views. Structural metrics are purely based on user interactions and, thus, provide limited information on the reasons and motivations of the groups. To get a first assessment of the content discussed in each of the two opposed communities and evaluate if they have confronted perspectives on climate change, we inspect the patterns of hashtags shared. Despite their ideological confrontation, they share most of them, likely due to the common subject of discussion (electronic supplementary material, figure S15). The mainstream communities use rather generalist hashtags such as #COP27, #IPCC, #climatechange, #ar6 or #climatereport. In Doñana, common hashtags between communities are less frequent, which could indicate a stronger political polarization. We observe hashtags related to the environment such as #donana, #donanaseextingue or #salvemosdonana for the mainstream community and hashtags related to right-wing parties such as #teamvox, or against the left-wing government such as #gobiernodimision, in the right-wing biased community.

**Figure 5.**
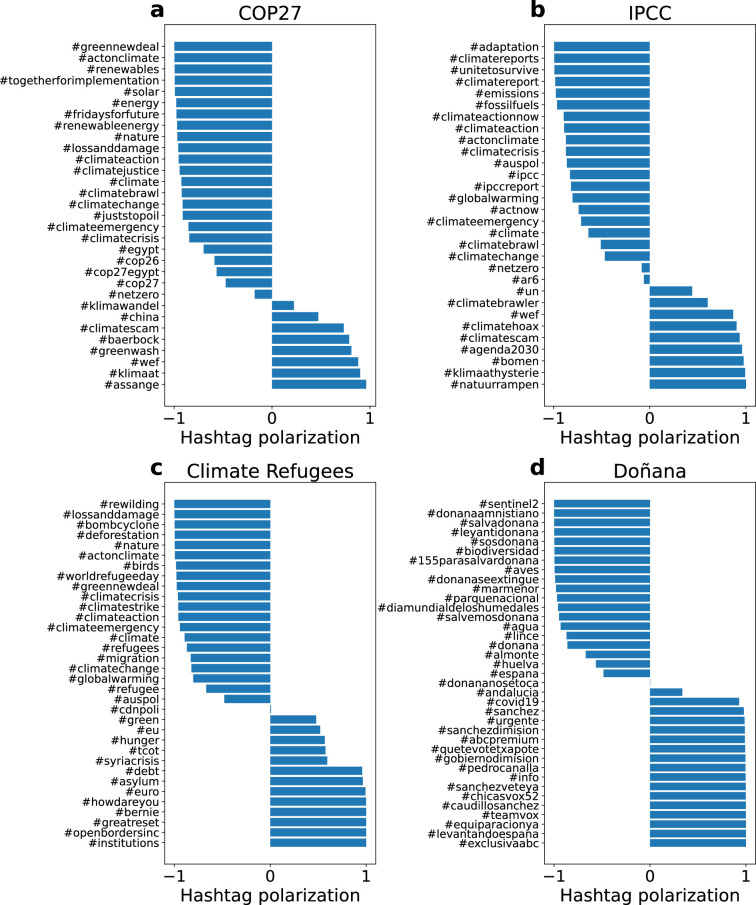
Hashtag polarization. Polarization around hashtags for (a) COP27, (b) IPCC, (c) Climate Refugees and (d) Doñana. Negative and positive values correspond to hashtags more abundant in the left-biased and right-biased communities, respectively.

To compute the hashtag polarization, we calculated the fraction of tweets with each hashtag by community, given by


(3.4) $$P_{i}^{\#}=\frac{S^{l}N_{i}^{r}-S^{r}N_{i}^{l}}{S^{r}N_{i}^{l}+S^{l}N_{i}^{r}},$$

where $N_{i}^{l}$ and $N_{i}^{r}$ are the number of tweets with the hashtag *i* in the main left- and right-wing communities, respectively, and $S^{l}$ and $S^{r}$ the number of users in each community. This formulation takes into account the uneven sizes of the communities. We display the polarization of the top hashtags in figure 5. The number of hashtags in each network varies since we have considered the 20 most shared in each community, and some of them can overlap. Interestingly, we observe only a few hashtags with values close to 0, suggesting a strong polarization in the use of hashtags. In the IPCC and COP27 datasets, the mainstream community dominates most of the hashtags, while the community spreading a counter-narrative dominates only a few hashtags related to the denial of climate change that include #climatehoax or #agenda2030. In COP27, we also observe hashtags related to environmental hypocrisy such as #greenwash. In the Doñana network, we also observe only a few hashtags close to zero and hashtags almost equally divided between +1 and −1. We performed a *Z*-test to assess the significance of hashtag frequency differences between the most polarized communities. Among the hashtags with the highest absolute *Z*-scores, #climatescam is predominantly used by right-leaning communities in both the COP27 and IPCC networks. In the Climate Refugees network, one of the hashtags with a high *Z*-score is #howdareyou, again primarily used by the right-wing community. In the case of the Spanish discussion about Doñana, the hashtags with the highest *Z*-scores are those criticizing the Spanish government, as previously mentioned (see electronic supplementary material, §S3.1, for details).

Whereas hashtags can provide a glimpse of the ideas discussed within each community, they offer a limited perspective to the opinion of individuals as they can be used in different contexts. To better understand the divergence in climate change views, we conducted a topic analysis on tweets from the two largest communities with opposing political perspectives. We assess topic polarization by quantifying the number of users within each community who discuss a given topic, using equation (3.4). Most topics are either dominated by the left-biased community or the low-reliability community. In the COP27 network, the right-leaning discourse reveals corruption discussions with money-related terms and political references to the UK, China and India. In contrast, the left-biased community focuses on essential resources such as water and food, as well as the urgency of taking action. In the IPCC network, topics within the right-biased community express scepticism about the scientific basis of climate change predictions, reference COVID-19, and include discussions that are often unrelated or misleading (for more details about the topics, see electronic supplementary material, §S3.2). The results confirm that it could be composed of individuals posting low-reliability information. The left-biased community emphasizes the need to reduce emissions and the urgency of taking action, using language similar to that found in COP27 discussions. In the Climate Refugees network, the right-biased community focuses on jobs and border security, whereas the left-biased community highlights the risks posed by natural disasters. The greatest divergence in discussions is observed in the Doñana network. The right-biased community focuses on the political handling of the situation, with an emphasis on the Spanish president, the ruling party (PSOE) and the European Union. In contrast, the mainstream community centres its discussion on the Doñana National Park and its conservation.

**Figure 6.**
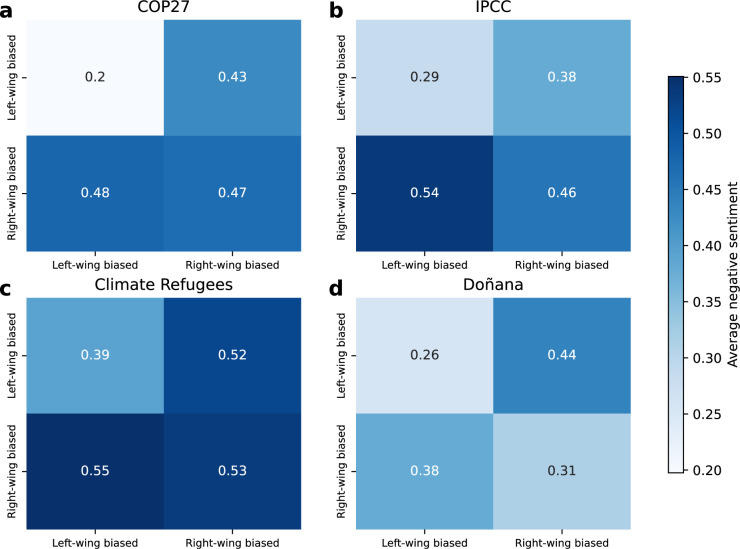
Average negative sentiment in messages in community interactions. Average negative sentiment of the messages as a function of the poster community (*y*-axis) and quoted (*x*-axis). The results correspond to (a) COP27, (b) IPCC, (c) Climate Refugees and (d) Doñana.

Besides the disparity in the content shared, via hashtags and topics, we want to address whether those groups are aware of each other and if they are emotionally aligned. Therefore, we evaluate emotional repulsion in relation to political views by analysing the sentiment of quote messages. Specifically, we assess the average negative sentiment based on the community of both the poster and the quoter (figure 6). We have implemented a sentiment analysis on the four Twitter datasets using a RoBERTa-based model fine-tuned on around 58 million tweets [76,77]. Overall, messages from right-wing biased communities exhibit a higher degree of negative sentiment, particularly when directed at left-wing biased communities (0.48 for COP27, 0.54 for IPCC and 0.55 for Climate Refugees). The difference between intra- and inter-community messages is most pronounced in the IPCC and Doñana networks. In the IPCC network, the negativity score for right-leaning to left-leaning interactions is 0.54, while for right-leaning to right-leaning interactions, it is 0.46 (Δ=0.08). Similarly, in the Doñana network, right-leaning to left-leaning interactions have a negativity score of 0.38, compared to 0.31 for right-leaning to right-leaning interactions (Δ=0.07). Interestingly, the most pronounced difference in sentiment between intra- and inter-community interactions occurs among users in the left-wing biased community. In the extreme case of the COP27 network, left-leaning users display a Δ=0.23 difference, while also being associated with the dissemination of more reliable information. Across all networks, the lowest levels of negative sentiment are observed within the left-wing biased communities, ranging from 0.20 in COP27 to 0.39 in Climate Refugees. In contrast, the right-wing biased community exhibits consistently higher negativity, even within its own interactions, with values ranging from 0.31 in Doñana to 0.53 in Climate Refugees.

We also conducted a Mann–Whitney *U*-test to evaluate the statistical significance of negativity differences between communities. A consistent pattern emerges in the COP27, IPCC and Climate Refugees networks: right-leaning to left-leaning interactions display significantly greater negativity than all other combinations. For example, in the IPCC network, the negativity of right-leaning to left-leaning interactions is significantly greater than that of left-wing biased to right-wing biased interactions (p<0.001), left-wing biased to left-wing biased interactions (p<0.001), and right-wing biased to right-wing biased interactions (p<0.001) (see electronic supplementary material, figure S19, for additional comparisons). Furthermore, across all datasets, right-wing biased to right-wing biased interactions exhibit significantly higher negativity compared to left-wing biased to left-wing biased interactions (COP27: p<0.001; IPCC: p<0.001; Climate Refugees: p<0.001; and Doñana: p<0.001). A similar pattern is observed when comparing left-wing biased to right-wing biased interactions against left-wing biased to left-wing biased interactions (COP27: p<0.001; IPCC: p<0.001; Climate Refugees: p<0.001 and Doñana: p<0.001). Hence, the structural communities identified are aligned with a divergence on content discussion related to climate change and stronger emotional responses. The results suggest that polarization on the subject takes multiple forms.

Three of the co-authors of the paper have conducted a manual annotation and review of a subset of the inter-community messages to validate the results of the sentiment detection framework (see electronic supplementary material, §S3.3). We computed the weighted Cohen’s Kappa coefficient and the interclass correlation coefficient to assess agreement among the three reviewers and between the reviewers and the model. All metrics indicate a moderate agreement between reviewers and between the reviewers and the model. Moreover, the correlations between the reviewers and with the model are aligned, suggesting that model scores fall within the range of variation observed among human annotators. We provide the annotated dataset, which includes the text, reviewer scores and model scores in [78].

### 3.4. Cross-platform analysis

We have analysed the dataset created from the YouTube videos quoted and referenced on Twitter to assess if the observed polarization facets are aligned with content consumption in video platforms as partisan media affects the dynamics of social networks [79]. The information gathered for each post includes the transcription and description of each video, the corresponding channel, and the user comments. The comment information allows us to create a bipartite network between users and posts since we know the users who have commented on each video. We can project the bipartite network into a post network or a user network. In the post network, the weight corresponds to the number of users in common and, in the user network, to the posts in common. The graphs are undirected by construction in both cases. We performed a community analysis using the greedy modularity optimization on the weighted network [69]. The organization of communities does not display a clear separation between communities with several interconnections, except for the Doñana network, where communities 0 and 1 have a clear separation (see electronic supplementary material, §S4).

We conducted an analysis linking Twitter and YouTube social media platforms to study the cross-platform effects. We create the Twitter subnetwork of users who shared YouTube links related to climate change, tagged according to the YouTube community of the videos. The connection between network organization and the shared YouTube content is clearer in the Doñana and IPCC networks. In both cases, users tweeting videos from YouTube community 0 are more tightly connected than other users (see the subnetwork in electronic supplementary material, figures S22 and S23).

Furthermore, we computed the frequency with which each Twitter community references YouTube posts and their corresponding communities. To account for the heterogeneity in community sizes observed in the YouTube dataset, we normalized the number of references to each YouTube community by its size. A strong connection exists between Twitter and YouTube communities, as most references within each Twitter community align with a specific YouTube community. Additionally, each Twitter community exhibits a unique pattern of references to YouTube.

**Figure 7.**
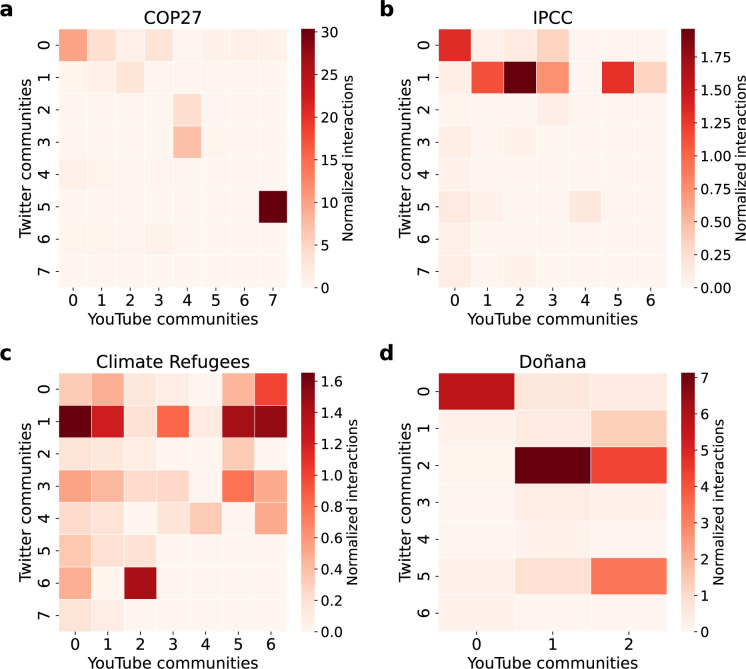
Interaction between Twitter and YouTube communities. Number of references to YouTube communities by the top six Twitter communities in (a) COP27, (b) IPCC, (c) Climate Refugees and (d) Doñana. To account for the uneven size of YouTube communities, we have divided the counts by the YouTube community size.

In the COP27 network, the larger left-wing biased Twitter community 0 predominantly references YouTube community 0 (35.8%), while the larger right-wing biased and low-reliability community 1 primarily references posts in YouTube community 2 (57.3%). Similarly, in the Climate Refugees network, Twitter community 6, known for spreading low-reliability information, interacts significantly with YouTube community 2 (74.6%). Finally, in the Doñana network, community 0 from both social networks interacts intensively (84.3%). Conversely, in the IPCC network, the larger right-wing biased community 1 references multiple YouTube communities, specifically communities 1 (20.2%), 2 (35.3%) and 5 (23.4%), suggesting a tendency to share diverse typologies of YouTube content. With the exception of the IPCC network, posts shared by the larger right-wing biased community on Twitter predominantly align with a single YouTube community (figure 7).

We have conducted a word frequency and topic analysis within the two YouTube communities with the most references from the main opposed Twitter communities (see electronic supplementary material, §S4). We observed an alignment between the topics discussed on Twitter and YouTube. Whereas the YouTube content connected to the Twitter mainstream community is more centred on the environment, the content connected to the low-reliability community has more references to models, data and scientists. An observation that was already noted in the topic analysis conducted on Twitter. The differences are more pronounced in the Climate Refugees and Doñana datasets. In the Climate Refugees dataset, mainstream content focuses on natural disasters, whereas the low-reliability community discusses refugees seeking asylum in the United States. In Doñana, the left-biased mainstream community has references to the natural environment and the largest right-wing party, whereas the right-biased low-reliability community is centred on the president of Spain, who represents the largest left-wing party in the country.

## 4. Discussion

Our results confirm that polarization in climate change discussions manifests in different forms through structural, ideological and emotional components. We also observed distinct views on climate change and patterns of content consumption. The structural polarization around the subject is aligned with distinct ideological biases and information reliability consumption. Not only that, the topological communities also show an alignment on perspective towards the environmental crisis and a stronger emotional response. Overall, polarization takes multiple aligned forms. While previous work often emphasized the role of ideological segregation, our cross-platform analysis shows that inter-community exposure remains significant—especially via mentions—but is frequently accompanied by affective repulsion and asymmetric negativity, particularly from low-reliability, right-leaning communities.

This suggests that polarization is not merely a consequence of echo chambers or algorithmic filtering, but also reflects active processes of antagonism and identity signalling, in line with theories of affective polarization. Even in multilingual and transnational contexts, such as the COP27 dataset, we observe stable polarization structures shaped more by ideological alignment and emotional tone than by language or geography. Moreover, the structural signature of interactions varies across platforms and interaction types: while retweets act as endorsement signals and tend to reinforce in-group cohesion, mentions frequently bridge ideological divides, albeit often with antagonistic or negative sentiment.

The interplay between information reliability and ideological alignment also emerges as a key factor. Right-leaning communities consistently share lower-reliability content and exhibit higher isolation and negativity, yet they remain engaged with mainstream communities—albeit often in confrontational terms. This pattern challenges the notion that low-reliability information spreads in isolation and points instead to a contested media space where ideological identity, trust and emotion are tightly intertwined. Our results align with other studies showing that, despite higher engagement within ideologically similar groups, users are still exposed to opposing views [17]. However, such exposure does not necessarily mitigate polarization. On the contrary, it has been shown that confrontation with opposing views can intensify polarization instead of reducing it [21]. This is reflected in our sentiment analysis, where messages from right-biased communities consistently show higher levels of negativity—especially when directed towards left-leaning users [60]. Overall, the sentiment in the low-reliability community was more negative. Our results are in line with recent research [26], which finds that climate change dissenters are closely related and tend to engage with a left-biased mainstream community [26,60,80]. Furthermore, toxicity levels are not evenly distributed across interaction types: replies and mentions are significantly more toxic than other forms of engagement [20]. This observation suggests that low-reliability individuals may reference the mainstream community to engage in discussions but get little attention in return [20], highlighting an asymmetry between left- and right-leaning communities [81].

The cross-platform alignment between Twitter and YouTube reinforces the idea that ideological communities are transversal across platforms, with users not only engaging with like-minded individuals but also selecting and amplifying content from ideologically aligned sources elsewhere. This calls for a broader, integrated approach to studying online polarization, moving beyond single-platform analyses. Additionally, our results indicate that the degree and manifestation of polarization can vary substantially across topics and events—suggesting that it is not static, but shaped by contextual factors such as the political salience of the issue or the type of event. This may reflect a dynamic of ideological sorting, whereby individuals increasingly align their social media behaviour with broader political identities, as already observed in the Finnish Twittersphere [48]. Prior studies, such as [54], observed that the 2019 IPCC Special Report on Climate Change and Land sparked significant public discourse on social media, with dietary recommendations becoming a highly polarized topic. Similar to our findings on localized polarization patterns (e.g. Doñana), that report showed that climate solutions affecting lifestyle choices may trigger elevated levels of contention on platforms like Twitter. The alignment between platforms could be amplified by recommendation algorithms, which can function as filter bubbles that reinforce users’ beliefs [13,82]. However, recent studies have downplayed the contribution of algorithms to polarization [83]. Our results support the idea that polarization is ubiquitous across platforms, but not driven solely by lack of exposure to opposing viewpoints.

These findings have important implications for the broader climate change debate. The persistence of polarized, emotionally charged interactions—even in the presence of exposure to opposing views—suggests that efforts to foster consensus around climate policy must account for the affective and ideologically driven nature of public discourse. Moreover, the asymmetric structure of negativity and the association between ideological alignment and information reliability can contribute to public confusion, distrust in science and a fragmentation of climate narratives, making it harder to establish the shared understanding necessary for effective action. Addressing polarization in this context requires more than correcting low-reliability information—it demands strategies that bridge ideological divides and rebuild trust in both institutions and science.

Our static analysis of the polarization around climate change could be extended to assess how it evolves with time as a function of internal dynamics, external sources and the unfolding of events. For instance, coordinated campaigns have been observed to impact social media dynamics [84] and could play a role in shaping the emergence or intensification of polarization. It is unclear whether message negativity is intrinsic to the topic or evolves with the discussion, potentially triggered by specific interactions. The asymmetric spread of emotionally polarized content suggests that by targeting specific small groups, polarization could be significantly decreased [80]. However, the opacity of most online social networks makes content moderation a major challenge. It would be essential to assess whether content moderation or fact-checking could have a positive impact on the reduction of polarization. Since our results suggest that individuals sharing low-reliability information are exposed to opposing views, the development of intervention strategies becomes a major challenge. The role of exposure in increasing polarization could be further addressed by investigating the temporal evolution of user ideology and reliability as a function of their progressive interactions with others. In a similar fashion, it opens the door to the development of models to unveil how the exposure to opposing views can further exacerbate polarization.

## Data Availability

The Twitter data are made available in accordance with Twitter’s terms of service at https://github.com/clint-project/clint-data [85] and the codes to calculate the metrics at https://github.com/clint-project/polarization_metrics [86]. The YouTube data are available at Zenodo [87]. The annotated dataset is available at Zenodo [78]. Electronic supplementary material is available online [88].
